# Myelodysplasia in the setting of paroxysmal nocturnal hemoglobinuria: Interpretation of blast percentage in a marrow with erythroid hyperplasia

**DOI:** 10.1002/jha2.37

**Published:** 2020-08-31

**Authors:** Emma M. Groarke, Bhavisha A. Patel, Alina Dulau-Florea, Irina Maric, Neal S. Young, Katherine R. Calvo

**Affiliations:** 1 Hematology Branch, National Heart Lung and Blood Institute, National Institutes of Health, Bethesda, Maryland, United States of America; 2 Hematology Section, Department of Laboratory Medicine, Clinical Center, National Institutes of Health, Bethesda, Maryland, United States of America


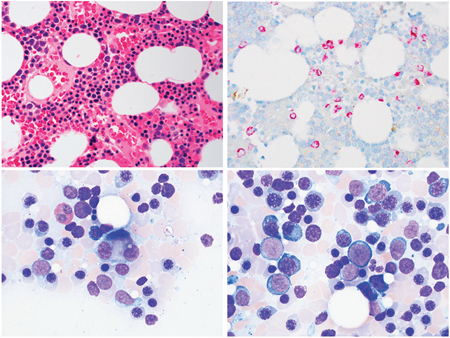


Severe aplastic anemia (SAA) is a bone marrow failure syndrome occurs most commonly due to immune destruction of the marrow stem cell. Paroxysmal nocturnal haemoglobinuria (PNH) is associated in many cases with SAA and can exist as a clinically insignificant low-level clone or with overt hemolysis and other clinical sequelae. Standard treatment of SAA is with either hematopoietic stem cell transplant (HSCT) or immunosuppressive therapy (IST). Those treated with IST incur a lifetime risk of clonal evolution to a myeloid neoplasm of approximately 10–15%. Here, we present a case of clonal evolution that posed a significant initial diagnostic challenge compounded by concurrent PNH.

A 64-year-old male with a diagnosis of SAA and PNH treated with immunosuppression 5 years prior and on eculizumab for treatment of a large PNH clone presented for his yearly surveillance visit clinically well and with bloodwork unchanged from previous year (Hb 12.5 g/dL, platelets 115 K/*μ*L, and neutrophils 1.92 K/*μ*L). PNH clone of 81.9% of glycosylphosphatidylinositol (GPI) negative neutrophils was detected. Routine marrow performed was normocellular with no morphological dysplasia. Aspirate total cell differential showed a blast count of 3% with a reversed M:E ratio of 1:2. Marrow biopsy immunohistochemistry (IHC) for CD34 was positive in 4-5% of all nucleated cells. Flow cytometry showed 2.4% myeloblasts (CD34+, CD13+, CD33dim+, CD117partial+, CD38+, and HLA-DR+); a small subset also consisted of CD123+, suggesting dendritic cell differentiation. MPO and TdT were not performed. There were no early B-cell precursors by flow. Cytogenetics showed expansion of a previously documented -Y (45,X,-Y[7]/46,XY[3], -Y in 7 of 10 metaphases). Standard next-generation sequencing (NGS) of a 177 myeloid cancer gene panel from peripheral blood was negative.

Although WHO criteria for a myeloid neoplasm were not met, close observation was undertaken due to the borderline increase in blast count. Six weeks later blood counts declined to Hb 10.4 g/dL, platelets 50 K/*μ*L, and neutrophils 1.17 K/*μ*L with a normal reticulocyte count. Repeat marrow was normocellular (top left image, medium power, H&E stain) and IHC for CD34 showed 5-6% positive cells of all nucleated cells (top right image, medium power). Megakaryocytic dysplasia was evident (bottom left image, high power, aspirate). M:E ratio was further reduced at less than 1:4. The aspirate cell differential showed 6% blasts in a background of marked erythroid hyperplasia and severe myeloid hypoplasia (bottom right image, high power, Wright-Giemsa stain). Flow cytometry showed 3.7% myeloblasts the majority of which had an aberrant phenotype; 50% coexpressed myeloperoxidase (MPO) and terminal deoxynucleotidyl transferase (TdT) and were negative for CD19, cCD79a, and cCD3, while 30% of myeloblasts were MPO and TdT negative but expressed CD123. Cytogenetics showed -Y in all 20 metaphases. Standard NGS remained negative. A diagnosis of myelodysplastic syndrome with excess blasts 1 (MDS-EB1) was made; the patient was treated with a hypomethylating agent and later underwent a successful hematopoietic stem cell transplant from a matched unrelated donor.

In cases with co-existing peripheral destruction of red cells, marrow erythroid hyperplasia may complicate recognition of an increased blast count in an evolving myeloid neoplasm. High normal or borderline blast counts may be significant and warrant close observation, particularly in patients with SAA who have an increased risk of both PNH and myeloid malignancy.

## Data Availability

The data that support the findings of this study are available from the corresponding author upon reasonable request.

