# Corrigendum

**DOI:** 10.1002/jha2.345

**Published:** 2021-11-24

In the article by Groarke, EM, Patel, BA, Dulau‐Florea, A, et al. [1], the authors would like to add the following funding source under funding information:

“National Heart, Lung, and Blood Institute and the National Institutes of Health Clinical Center.”
